# Translation and validation of the Dutch Pittsburgh Fatigability Scale for older adults

**DOI:** 10.1186/s12877-020-01630-8

**Published:** 2020-07-08

**Authors:** Marlies Feenstra, Nynke Smidt, Barbara C. van Munster, Nancy W. Glynn, Sophia E. de Rooij

**Affiliations:** 1 Department of Internal Medicine and Geriatrics, University of Groningen, University Medical Center Groningen, HPC: AA43, PO Box 30001, 9700 RB Groningen, The Netherlands; 2 Department of Epidemiology, University of Groningen, University Medical Center Groningen, Groningen, The Netherlands; 3 Department of Geriatrics, Gelre Hospitals, Apeldoorn, The Netherlands; 4 Department of Epidemiology, Graduate School of Public Health, Center for Aging and Population Health, University of Pittsburgh, Pittsburgh, PA USA; 5 Medical School Twente, Medical Spectrum Twente, Enschede, The Netherlands

**Keywords:** Fatigue, Fatigability, Psychometric properties, Validity, Reliability, Geriatric medicine

## Abstract

**Background:**

The original Pittsburgh Fatigability Scale (PFS) was developed to assess perceived fatigability in older adults. The objective of this study was to translate the PFS into Dutch and investigate its validity and reliability among hospitalized older adults aged ≥70 years.

**Methods:**

The PFS was translated into Dutch and pretested for comprehensibility by the Three-Step Test Interview method. The factor structure underlying the final version was evaluated by confirmatory factor analysis (CFA) and exploratory factor analyses (EFA). Internal consistency of the identified subscales was evaluated by Cronbach’s alpha. Construct validity was evaluated by hypothesis testing. Test-retest reliability was evaluated using intraclass correlation coefficients (ICC) and Bland Altman plots.

**Results:**

The validation sample included 233 patients. CFA of the original factor structure resulted in poor model fit in our Dutch sample. EFA of PFS physical and mental subscales resulted in a two-factor solution underlying the data with good internal consistency of the identified subscales (Cronbach’s alpha: 0.80–0.92). Five out of six hypotheses were confirmed, indicating good construct validity. Retest assessments were performed among 50 patients and showed good reliability for both the physical (ICC: 0.80, 95%CI: 0.68; 0.88) and mental subscale (ICC: 0.81, 95%CI: 0.68; 0.89).

**Conclusion:**

The Dutch PFS is a valid and reliable instrument to assess fatigability in older hospitalized patients.

## Background

Fatigue is a common burdening symptom among older adults that affects well-being [1]. Older adults often perceive fatigue without a clear physiological cause, which is suggested to be a consequence of aging in general [2, 3]. Prevalence rates of fatigue vary from 28 to 55% in adults aged 65 years and older and from 68 to 87% among the 85-year olds depending on the measurement tool [2, 4]. Fatigue in older adults is associated with persistent functional decline [5, 6] and is suggested to be a clinical marker for identifying persons at risk for negative health outcomes, such as frailty, disability, and hospitalizations [2, 3, 7].

There are many well-developed and validated uni-or multidimensional instruments to assess fatigue, such as the Brief Fatigue Inventory, Multidimensional Fatigue Symptom Inventory or the Fatigue Severity Scale [8, 9]. However, a key limitation of these tools is that they do not take the activity level of the respondent into account, potentially leading to information bias. For instance, sedentary people may perceive lower fatigue levels than their more active counterparts, making their outcomes hard to compare and interpret. Fatigability, defined as the measurement of fatigue in relation to an activity of fixed intensity and duration, accounts for this so called self-pacing bias [7, 10]. Thus, fatigability is a more sensitive measure of someone’s susceptibility to fatigue, making this construct more suitable as a research outcome [7, 11, 12].

Fatigability can be assessed using performance-based or self-report tools [10, 13]. Performance fatigability is measured by the change in performance (e.g. slowing down during a physical performance test such as a 400 m walk) without asking for the perception of fatigue [10, 14]. Self-reported or perceived fatigability can be measured in two ways: 1) self-reported fatigue after performing an activity, and 2) asking for the expected fatigue after performing activities of a certain intensity and duration, without actually performing the activity [7, 14, 15]. The latter is especially suitable for populations in which performing activities is too challenging, such as hospitalized, bedridden older adults, or for large scale studies where equipment or space is not available [15]. A few self-administered instruments are available that attempt to measure fatigability: Mobility Tiredness Scale [16], Dutch Exertion Fatigue Scale [17], Situational Fatigue Scale [18], and the Pittsburgh Fatigability Scale (PFS) [15]. Both the Mobility Tiredness Scale and the Dutch Exertion Fatigue Scale were developed for the assessment of fatigue during activities of daily living [16, 17]. The first was developed for older people, the latter for patients with heart failure. However, the items of both instruments do not consistently refer to the intensity and duration of every activity, and making them prone to self-pacing bias. The Situational Fatigue Scale included items referring to both physical and mental fatigability using items referring to activities with fixed intensity and duration, but was developed for and validated in younger age groups [18]. The PFS was specifically developed for the assessment of both physical and mental fatigability in older people and consists of multiple items that describe activities of varying duration and intensity [13, 15]. The PFS showed good convergent validity against measures of performance fatigability in older American adults [15], and its predictive validity for mobility decline has recently been demonstrated [19]. Considering these aspects, together with the fact that, in Dutch, an instrument to measure perceived fatigability that is suitable for older adults is still lacking, the purpose of this study was to translate the PFS into Dutch and subsequently investigate its validity and reliability among Dutch hospitalized older adults.

## Methods

### Study design and patient selection

A prospective cohort of patients aged 70 years and older admitted to the University Medical Center of Groningen (UMCG) were recruited from April 2018 to April 2019. Both acute and elective patients admitted to cardiology, oncology, vascular and hepatobiliary, trauma, and internal medicine units were screened for participation every weekday. Inclusion criteria were age 70 years and older and an expected hospital stay of at least two consecutive days. Exclusion criteria were: no understanding of the Dutch spoken language, any (temporary) cognitive condition that influence decision making capacity, and no written informed consent. Enrollment took place within the first 4 days of hospital admission. Baseline questionnaire data included demographics, activities of daily living, cognitive functioning, the Frailty Phenotype, and the PFS. To assess reliability, a random selection of the included patients were invited for a second assessment of the PFS 1 day after their baseline assessment. No surgical procedures were conducted between test and retest assessments. After hospital discharge, medical charts were consulted to measure comorbidity status and type of hospital admission and treatment (acute, elective, non-surgical, surgical). Data were collected by trained research staff of the geriatric medicine department at the UMCG.

### Translation procedure of the PFS

The PFS was originally developed in the United States to measure perceived physical and mental fatigability for adults aged 60 years and older. The PFS consists of ten items describing an activity with a certain intensity and duration. For each item, three questions are formulated: first the expected or imagined physical fatigue immediately after completing the listed activity; second the expected or imagined mental fatigue immediately after completing the listed activity; and third, whether or not the activity was done in the past month. For each item, physical and mental fatigue are rated on a scale from 0 to 5, where 0 represents no fatigue and 5 represents extreme fatigue. Scoring results in a physical fatigability score and a mental fatigability score, each ranging from 0 to 50 points, with higher scores indicating greater fatigability. The original questionnaire was validated for the PFS physical subscale, which yielded four factors: 1. moderate to high intensity activities, 2. social activities, 3. sedentary activities, and 4. lifestyle or light intensity activities [15].

The PFS was translated into Dutch according to a formal forward/backward translation protocol [20]. Briefly, the following stages were completed: [1] forward translation by two independent bilingual, native Dutch speaking translators [2]; synthesis of discrepancies of the two translated versions into one translated version by the translators and an observant [3]; back translation by two independent bilingual, native English speaking translators, who were blinded for the original version of the PFS [4]; review and syntheses of the two translated versions by an expert panel consisting of the original developer, a linguistic, the translators, and a geriatrician [5]; field testing of the pre-final version among the target population. The field test included conducting interviews using the Three-Step Test Interview method in a small sample of the target population to assess comprehensibility of the Dutch PFS [21, 22].

### Questionnaires

Concurrent with the PFS, patient characteristic and questionnaire data were ascertained for the purpose of descriptive statistics and construct validity. Demographic characteristics included age, sex, and level of education (low (<high school), moderate (high school), high (college / university) [23], all measured via self-report questionnaires. Clinical characteristics included the Charlson Comorbidity Index (CCI) [24] and length of hospital stay (LoS), both obtained from medical records.

Pre-hospital existing problems with performing instrumental activities of daily living (iADL) were assessed using the seven original Lawton and Brody items including the use of a walking aid [25]. Patients were asked to refer to the situation 2 weeks before hospital admission. Total scores ranged from 0 to 8 with higher scores indicating more limitations in iADL.

Pre-hospital frailty status was assessed using the Frailty Phenotype based on self-report [26, 27]. Unintended weight loss was considered when patients reported losing three or more kilograms of body weight in the past month. The items exhaustion, slowness, weakness, and low physical activity referred to the situation 2 weeks before hospital admission. Exhaustion was assumed when people scored ‘yes’ to both questions, ‘everything I did was an effort’, and ‘I could not get going’ [28]. Slowness was assumed when people were not able to walk outside for 5 min. Weakness was assumed when people reported to have difficulties rising a chair. Low physical activity was assigned when people reported not being physically active for at least 30 min per week. Total scores ranged from 0 to 5, with higher scores indicating more frailty.

Cognitive functioning was assessed using the Short Blessed Test (SBT) [29]. The SBT included three questions addressing orientation: two items addressing attention and one item addressing memory. A weighted scoring was used ranging from 0 to 28 points, with higher scores indicating more cognitive impairment.

### Statistical analysis

#### Descriptive statistics

For all baseline demographic and clinical characteristics, descriptive statistics were calculated as mean (standard deviation), median (inter-quartile range), or N (%).

#### Validity

Construct validity, defined as the degree to which scores of a measurement are consistent with hypotheses, was assessed by hypotheses testing and factor analyses.

1. Hypothesis testing was performed using iADL and physical frailty as related dissimilar constructs and cognitive functioning as an unrelated construct. In total six hypotheses were formulated:
  - The PFS physical score has a moderate positive correlation (*r* = 0.3–0.5) with the sum score of the iADL items.
  - The PFS physical score has a moderate positive correlation (*r* = 0.3–0.5) with the Frailty Phenotype.
  - The PFS mental score has a moderate positive correlation (*r* = 0.3–0.5) with the sum score of the iADL items.
  - The PFS mental score has a moderate positive correlation (*r* = 0.3–0.5) with the Frailty Phenotype.
  - The PFS physical score has a low correlation (*r* ≤ 0.3) with the sum score of the SBT.
  - The PFS mental score has a low correlation (*r* ≤ 0.3) with the sum score of the SBT.

Pearson’s Correlation Coefficients or Spearman’s Rank Correlation Coefficients were used based on normal or non-normal data distribution, respectively. Construct validity of the PFS was considered to be good if at least five hypotheses were confirmed [30].

2. Confirmatory Factor Analysis was utilized to investigate if the factor structure of the original PFS physical subscale was also found in the Dutch sample. To evaluate model fit, standardized root mean square residual (SRMR ≤0.08) and comparative fit index (CFI ≥ 0.95) were used [31].
3. Exploratory Factor Analyses (EFA) of the PFS physical and mental subscales were performed to explore the best factor structure underlying the Dutch sample. We followed the procedure suggested by Osborne & Ostelo (2005) [32], which is explained in detail in Additional File 1. A good fit was considered when the items within the factor had all loadings > 0.3, but preferably > 0.5, and no or few items existed with cross-loadings > 0.32 [32]. Finally, the interrelatedness of the items within each factor of the best-fitted factor solution was examined by Cronbach’s alpha. Cronbach’s alpha of 0.70 or higher was considered appropriate [33].

#### Reliability

Reliability, defined as the extent to which the measure is free of measurement error, was assessed for both the PFS physical and mental scores of a random subsample who gave consent to a second PFS assessment 1 day after the baseline assessment.

1. Test-retest reliability was calculated with an intraclass correlation coefficient (ICC) using a two-way mixed effects model for absolute agreement. Cut-off values for interpretation of the ICC were < 0.5 poor, ≥0.5 and < 0.75 moderate, ≥0.75 and < 0.9 good, ≥0.9 excellent reliability [34].
2. Agreement, defined as the ability of the instrument to result in the same scores when applied twice under the same conditions, was assessed by calculating Bland-Altman plots. Therefore, mean change scores of baseline and retest assessments were plotted against the difference of both scores. Paired t-tests were calculated to assess systematic measurement error [33, 35].
3. Measurement error was calculated by the standard error of measurement (SEM) using the following formula: SEM = SD(T0) x √(1-r). SD(T0) refers to the standard deviation of the baseline measurement; r refers to the ICC calculated in step 1. To interpret the SEM, scores were converted to percentages of the scale range and cut-off values were used as suggested by Ostelo and colleagues (2004) [36]: ≤5% very good, ≤10% good, > 10 and < 20% moderate, ≥20% poor.
4. Smallest detectable change (SDC) defined as the smallest unit of change that can be detected by the instrument beyond measurement error was calculated by the following formula: SDC = 1.96 x √2 x SEM. Both the absolute SDC value as well as the SDC as a percentage of the scale range were calculated.

#### Subgroup and sensitivity analysis

Construct validity was determined for both the total group, and a subgroup of elective and acute patients, and the subgroup of surgical and non-surgical patients. Data were imputed by item mean imputation using sample-specific correction factors for sex and activity level where three or less items of either the PFS physical or mental subscale were missing, but the related question on whether the activity had been done in the past month was completed [37]. Sensitivity analyses included validity and reliability analysis of patients with complete PFS data. Imputations and CFA analysis were performed in STATA SE, version 14. All other analyses were performed using Statistical Package for Social Science (SPSS) software, version 23.

## Results

### Translation of the PFS

During the forward translation of the PFS some language difficulties were identified, namely the translation of the answer category ‘extreme fatigue’ lead to discussion. Consensus was reached with assistance of a third independent person. The unit pounds (lbs.) (item g) is not used in the Dutch language and therefore needed to be converted into kilograms. The examples ‘senior center’, ‘hiking’, and ‘Zumba’ revealed translation problems, but consensus was reached by the translators. All solutions were approved by the members of the expert meeting. Twelve patients participated in the field test of the pre-final version. Patients sometimes reported difficulties distinguishing between physical and mental fatigue. In general, we identified two types of patients: 1. Patients who distinguished physical from mental fatigue using different definitions for both constructs; 2. Patients who did not distinguish these two concepts. However, all interviewed patients mentioned that both concepts were somehow related to each other. To clarify the construct of mental fatigue to patients that requested, we stated that mental fatigability refers to fatigue of the mind, not fatigue of the body or muscles. Patients reported to understand the questions, so no further adjustments of the Dutch PFS were done. The final version of the Dutch PFS is available on request by dr. Nancy W. Glynn [38].

### Descriptive statistics

After the field test, the Dutch PFS was administered to 249 patients. Sixteen patients (6%) were excluded from the analysis, because they had more than three missing values for the PFS, leaving 233 consecutive patients in the analytic sample for validation of the PFS. A flowchart of the study participants is included in Additional File 2, Figure S2–1. A random sample of 50 patients were selected out of the validation sample for re-assessment of the PFS 1 day after the baseline assessment to evaluate reliability. The median age of patients was 76 years (IQR: 73; 81) and the majority of the patients were male (66%). Table 1 presents baseline demographic and clinical characteristics of the total study population and reliability sample.

**Table 1:** Baseline demographic and clinical characteristics of the study samples

Characteristic	Validation sample (***n*** = 233)	Reliability sample (***n*** = 50)
Age in years, median (IQR)	76 (73; 81)	76 (72; 82)
range	70–95	70–87
Sex, male	154 (66)	27 (54)
Educational level		
low (< high school)	61 (26)	13 (27)
moderate (high school)	84 (36)	23 (48)
high (college / university)	83 (36)	12 (25)
Medical specialty associated with hospital admission		
trauma and orthopedics	17 (7)	2 (4)
vascular and hepatobiliary	37 (16)	6 (12)
medical oncology	40 (17)	11 (22)
internal medicine	20 (9)	9 (18)
cardiology	114 (50)	18 (36)
Comorbidity^a^, median (IQR)	2 (1; 3)	2 (1; 3)
range	0–10	0–9
LoS in days, median (IQR)	4 (3; 8)	5 (4; 11)
range	1–37	2–31
Fatigability score		
physical, mean (SD)	23.7 (11.5)	30.3 (9.8)
mental, mean (SD)	14.9 (13.5)	24.4 (13.6)

All numbers are presented in n (%), unless indicated otherwise.

a. Comorbidity score was assessed using the Charlson Comorbidity Index.

Abbreviations: *IQR* Inter Quartile Range; *LoS* Length of Stay; *SD* Standard Deviation

### Construct validity

Spearman’s correlation coefficients between PFS subscales and the other constructs are presented in Table 2. Five out of the six hypotheses were confirmed. Confirmatory Factor Analysis of the original four-factor model of the PFS physical subscale resulted in poor model fit (SRMR: 0.29; CFI: 0.75). Kaiser-Meyer-Olkin values were 0.84 and 0.89 for PFS physical and mental subscales, respectively. Bartlett’s test of sphericity were > 0.001 for both subscales, and no item correlations were < 0.2 or > 0.9, indicating that EFA could be applied.

**Table 2:** Expected and observed correlations of the Dutch PFS physical and mental subscales with other constructs

	Spearman’s rank correlation			
Instrument (scale range)	PFS physical score (0–50)		PFS mental score (0–50)	
	Expected	Observed	Expected	Observed
iADL (0–8)	0.3–0.5	0.4	0.3–0.5	0.3
Frailty Phenotype (0–5)	0.3–0.5	0.6	0.3–0.5	0.4
SBT (0–28)	≤ 0.3	0.1	≤ 0.3	0.1

Abbreviations: *iADL* Instrumental Activities of Daily Living; *PFS* Pittsburgh Fatigability Scale; *SBT* Short Blessed Test

Data for the PFS physical and mental items were non-normally distributed [Additional File 2, Table S2–1a and 1b, respectively], therefore the principle axis factoring extraction method (PAF) was used, with oblique rotation to estimate the optimal factor structure underlying the Dutch data. Regarding the PFS physical subscale, first the preselected four-factor solution was estimated, resulting in the following eigenvalues and cumulative percentage explained variance: 4.796 (48.0%), 1.711 (65.1%), 0.799 (73.1%), 0.620 (79.3%). Four items did not load on their intended scales based on the factor structure underlying the original validation sample. The scree plot showed points of inflexion three factors, which was indicative for a two factor solution underlying the Dutch dataset. Therefore, PAF using oblique oblimin rotations for a two-factor, three-factor, and five-factor solution were performed as well. The three-factor and five-factor solutions did not result in a clean factor structure. The two-factor solution resulted in two factors of each five items with factor loadings of > 0.5 with two theoretically interpretable factors. The first factor included items a, b, d, g, and j and was named ‘activities requiring physical effort’. The second factor included items c, e, f, h, and i which we named ‘activities requiring low physical effort’. One cross-loading of 0.33 for item c (light household activity for 1 h) existed in the two-factor solution. Cronbach’s alpha analysis showed good relatedness of the items of factor 1 (α: 0.87), and factor 2 (α: 0.83). Regarding the PFS mental subscale, the scree plot indicated a two factor solution underlying the Dutch data, with two eigenvalues of 5.960 and 1.019 explaining 59.6 and 10.2% of the variance. The two-factor solution resulted in one factor including the items a, b, c, d, g, and j referring to ‘activities requiring physical effort’ and a second factor including the items e, f, h, and i referring to ‘activities requiring low physical effort’. All factor loadings were > 0.5, but again item c (light household activity for 1 h) loaded on both factors. The pattern matrices of the two-factor solutions of both the physical and mental subscales relative to the factors of the original PFS physical subscale are presented in Table 3.

**Table 3:** Exploratory Factor Analyses results: Pattern matrices of the Dutch PFS physical and mental subscales

		Physical subscale		Mental subscale	
Factor / Item description		Factor 1 (α: 0.87)^**a**^	Factor 2 (α: 0.83)^**a**^	Factor 1 (α: 0.92)^**b**^	Factor 2 (α: 0.80)^**b**^
**Moderate to high intensity activities**					
B	Brisk walking	0.800	–	0.984	–
D	Heavy gardening	0.657	–	0.699	–
G	Strength training	0.781	–	0.596	–
J	High intensity activity	0.832	–	0.525	–
**Lifestyle or light intensity activities**					
C	Household activity	0.325	0.562	0.504	0.345
A	Leisurely walk	0.602	–	0.913	–
**Social activities**					
H	Participate in social activity	–	0.806	–	0.850
I	Hosting social event	–	0.753	–	0.794
**Sedentary activities**					
E	Watching TV	–	0.667	–	0.606
F	Sitting quietly	–	0.596	–	0.546

Factors were extracted by Principle Axis Factor analysis and rotated by oblique oblimin rotation.

Only factor loadings > 0.3 are presented.

a. Cronbach’s alpha was calculated using items B, D, G, J, A (Factor 1); C, E, F, H, I (Factor 2).

b. Cronbach’s alpha was calculated using items B, C, D, G, J, A (Factor 1); E, F, H, I (Factor 2).

### Reliability

**Fig. 1:**
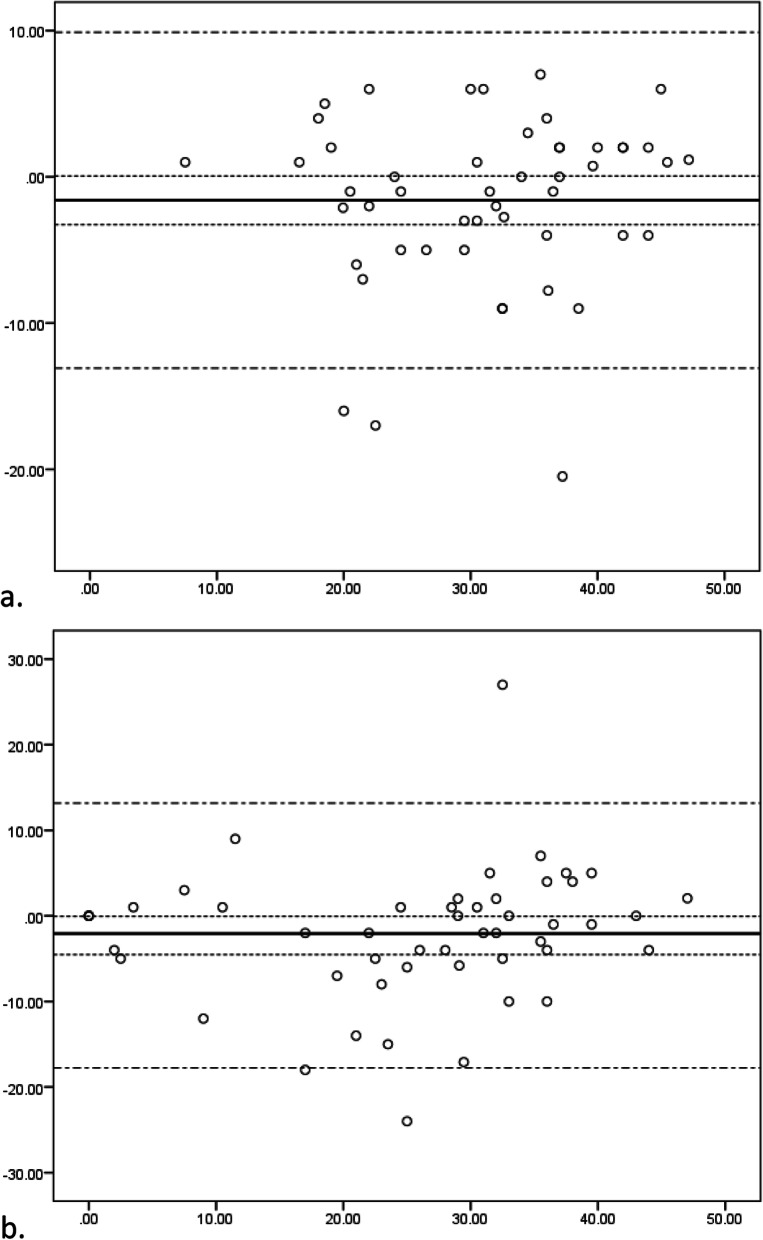
Bland Altman plots of the Dutch Pittsburgh Fatigability Scale physical (a) and mental (b) subscales. X-axis represent mean fatigability scores; Y-axis represents difference of fatigability scores (baseline – retest assessments)

Mean PFS physical and mental scores, ICC, SEM, and SDC are presented in Table 4. ICC of the PFS physical and mental subscales were 0.80 (95%CI: 0.68; 0.88) and 0.81 (95%CI: 0.68; 0.89), respectively, indicating good reliability. The Bland Altman plot showed good agreement for the PFS physical subscale (mean difference: -1.60, 95%CI: − 3.27; 0.06). The PFS mental subscale had a small mean systematic difference between baseline and re-assessment (mean difference: -2.30, 95% CI: − 4.54; − 0.05) (Fig. 1). SEM was good for the PFS physical subscale (9%), and moderate for the PFS mental subscale (12%) (Table 4). Smallest detectable change beyond measurement error was 12 (24%) and 16 (33%) points for the PFS physical and mental subscales, respectively (Table 4).

**Table 4:** Mean baseline and retest scores and reliability properties of the Dutch PFS (*n* = 50)

PFS	Baseline mean (SD)	Retest mean (SD)	ICC^**a**^ (95% CI)	SEM	SEM (%)^**b**^	SDC	SDC (%)^**b**^
Physical subscale	30 (10)	31 (10)	0.80 (0.68; 0.88)	4	9%	12	24%
Mental subscale	24 (14)	26 (13)	0.81 (0.68; 0.89)	6	12%	16	33%

Abbreviations: *ICC* Intra-class Correlation Coefficient; *PFS* Pittsburgh Fatigability Scale; *SD* Standard Deviation; *SDC* Smallest Detectable Change; *SEM* Standard Error of Measurement; *MIC* Minimal Important Change

a. A 2 way mixed effect model was used.

b. SEM (%) and SDC (%) are expressed in percentages of the scale range (0–50); percentages are rounded off.

### Subgroup and sensitivity analysis

We performed subgroup analyses of elective (*n* = 92) and acutely admitted (*n* = 97) patients as well as surgical (*n* = 142) and non-surgical patients (*n* = 60) [Additional file 3]. For all subgroups at least five out of six hypotheses were confirmed. EFA resulted in a two-factor solution for the PFS physical subscale for every subgroup. EFA of the mental subscale resulted in either a clean single factor solution (non-surgical patients), or a two factor solution (elective, acute, and surgical subgroups). The results of the sensitivity analysis using only data of patients with complete PFS data (*n* = 201) were generally comparable and did not lead to different conclusions [Additional file 3].

## Discussion

This study showed that the Dutch version of the Pittsburgh Fatigability Scale had good content validity, construct validity, and test-retest reliability for measuring perceived physical and mental fatigability in hospitalized adults aged 70 years and older.

The CFA analysis performed on the original four-factor solution of the PFS physical subscale resulted in poor model fit. A possible explanation for this finding was that the original validation study retrieved four factors from 26 initial items. During the development process, EFA was used to reduce the number of items resulting in the ten items included in the original PFS. The guideline provided by Osborne & Costello stated that factors with fewer than three items are likely to be unstable and should advocate to reduce the number of factors [32]. However, the original sample had a high subject to item ratio of 18:1 and all items had strong loadings on their corresponding factor (≥0.7), which is an indication of strong factors despite the low number of items per factor [32]. The two-factor solution found in the current data may have been appropriate in the original sample as well, but this was not reported. In the current data, a clean two-factor structure was found. However, one cross-loading existed for the item ‘light to moderate household activities for 1 hour’. This cross-loading existed in both the main analysis and all subgroup analyses, which could indicate an ambiguous translation. The predefined examples of light household activities included ‘cleaning, cooking, dusting, straightening up, baking, making beds, dishwashing, and watering plants’. There may have been confusion regarding the interpretation of the intensity of the example activities. It is plausible that patients may have interpreted cleaning as mopping or vacuuming, which has a higher intensity than for instance dusting. Moreover, cooking requires more mental and cognitive processes, such as planning and sequencing, than for instance dishwashing. Patients could have interpreted cooking as an activity that required more mental than physical effort, which may explain its cross-loading on the second factor referring to activities that require less physical effort. Lastly, men could have interpreted household activities differently from women, which could have led to the cross-loading found for household activities in the current dataset.

In the current study, mean fatigability scores were on average higher compared to mean fatigability scores of the original validation study [15]. This is not surprising given the sample was drawn from hospitalized older adults. Nonetheless, supplemental analyses comparing elective and acute patients showed that PFS scores were lower for elective patients. These patients are more likely to be representative of the general population as their fatigability scores were comparable to community dwelling Spanish older adults within the same age group [39] and community dwelling Americans with comparable number of comorbidities [40]. The highest fatigability scores were found in acute and non-surgical patients, which may be attributed to the illness and subsequent disability onset in the weeks before hospital admission that is commonly seen in older medical patients [41]. This explanation seems plausible, because in the Dutch sample we also found lower proportions of patients who performed activities that required physical effort in the past month compared to the original validation sample [15]. Exception to this was the proportion of patients that performed a brisk or fast walk in the previous month. In our study, 29% of the patients reported to have done a brisk or fast walk for 1 h compared to 17% of the older US adults in the original validation study [15]. This difference was unexpected, but likely explained by cross-cultural differences. Like Americans are known for their hiking culture [42], Dutch are known for their cycling culture [43]. Therefore it could be that our Dutch sample had difficulty distinguishing between a brisk and leisurely walk as walking is less culturally embedded.

Test-retest reliability was assessed using a measurement interval of 1 day to minimize the risk that conditions of the patients changed. The target population, hospitalized older persons, may fluctuate over time concerning illness and symptoms. The risk of recall bias has been considered, however it was expected that patients did not remember their initial answers, partly due to stressful circumstances patients are exposed to during hospitalization and because the first PFS assessment was part of a larger set of questionnaires assessed at the same time. Even though we took a random sample of the validation sample to test reliability, the reliability sample had higher initial PFS scores compared to the validation sample. As a result, there were few observations at the beginning of the scale range of corresponding Bland Altman plots, especially for the PFS physical subscale. However, the Bland Altman plots showed a homogeneous spread over the scale-range, supporting good agreement between the test and retest of both the PFS physical and mental subscales.

### Strengths and limitations

This is the first study that translated the PFS into Dutch. Strengths of this study include the relative large sample size as we included more than twice the recommended sample for validation studies [33]. A second strength is the exhaustive translation methodology including pretesting of the pre-final version [20]. There were limitations as well. First, this study was performed in one hospital, so there was risk of selection bias. Considering the fact that the UMCG provides both primary and specialist care for patients from several regions of the Netherlands, together with the broad inclusion criteria including patients from both medical and surgical wards, we have evaluated the selection bias as low. However, the robustness of the Dutch PFS should be further examined in other Dutch cohorts. Second, relative high missing values (7–8%) were present in the current study for the two items including high intensity activities. However, sensitivity analysis with only complete PFS data led to the same results, indicating robustness of our findings. Third, no convergent validity against performance-based fatigability assessment was performed due to lack of suitable measurement in this hospitalized population.

### Future research

The current study demonstrated that perceived physical and mental fatigability in Dutch hospitalized adults aged 70 years and over can now be assessed using the valid and reliable Pittsburgh Fatigability Scale. Future research should investigate the Dutch PFS’s validity and reliability in other Dutch samples like community-dwelling older adults. Further validation for the purpose of convergent validity of the PFS Dutch version physical subscale is recommended by using performance fatigability assessments, which have demonstrated to be feasible in older community-dwelling adults [9, 10, 33]. This is the first study that presented the factor solution of the PFS mental subscale, which in our population of hospitalized older adults indicated two factors underlying mental fatigability as the cleanest structure. Future research should demonstrate whether this factor structure also applies to other populations and subgroups. Furthermore, when the PFS is applied in cohort studies or as intervention outcome, future research should investigate its responsiveness and interpretability to examine the extent to which the PFS is able to measure change in fatigability over time.

## Conclusion

The translated Dutch version of the Pittsburgh Fatigability Scale is a valid and reliable instrument to assess perceived physical and mental fatigability in hospitalized adults aged 70 years and older.

## Supplementary information

**Additional file 1.** Exploratory Factor Analysis procedure. A detailed description of the procedure and decisions concerning Exploratory Factor Analysis.

**Additional file 2.** Supplementary figures and Tables. A flowchart of study participants and additional descriptive tables.

**Additional file 3.** Subgroup and sensitivity analyses. The results of the subgroup and sensitivity analyses.

## Data Availability

The datasets used during the current study are available from the corresponding author upon reasonable request.

## Abbreviations

ADL: Activities of daily living
CCI: Charlson comorbidity index
CFA: Confirmatory factor analysis
CFI: Comparative fit index
EFA: Exploratory factor analysis
iADL: Instrumental activities of daily living
ICC: Intra-class correlation coefficient
IQR: Interquartile range
LoS: Length of stay
PAF: Principle axis factoring extraction method
PFS: Pittsburgh fatigability scale
SBT: Short blessed test
SD: Standard deviation
SDC: Smallest detectable change
SEM: Standard error of measurement
SRMR: Standardized root mean square
UMCG: University medical center groningen
