# Supplementary material for: Translation and validation of the Dutch Pittsburgh Fatigability Scale for older adults
Source: BMC Geriatr. 2020 Jul 8;20:234. doi: 10.1186/s12877-020-01630-8 (PMC7346360; doi:10.1186/s12877-020-01630-8)
Supplement: Supplementary file 1 — Additional file 1. Exploratory Factor Analysis procedure. A detailed description of the procedure and decisions concerning Exploratory Factor Analysis. [file 12877_2020_1630_MOESM1_ESM.pdf]

**ADDITIONAL FILE 1: Exploratory Factor Analysis procedure**

Before starting the Exploratory Factor Analysis (EFA), suitability of the data for performing EFA was tested by the Kaiser-Meyer-Olkin test (≥0.8) and Bartlett's test of sphericity ($P<0.05$) (1). All EFA were performed using the procedure suggested by Osborne and Costello (2005) (2). First, the extraction and rotation methods were selected based on data characteristics. Concerning the extraction method, either maximum likelihood (ML) or principle axis factoring (PAF) extraction methods were used, depending on normal (ML) or non-normal (PAF) data distribution respectively. The rotation method should be selected based on the correlation between the factors: orthogonal rotations are applied to uncorrelated factors, oblique rotations are applied when factors correlate. In the current study, for all EFA analyses, direct oblique rotation was used to allow the factors to correlate. Second, the correlations between items were inspected. Items with correlations <0.20 with all other items were deleted immediately (3); items with correlations >0.90 might indicate multicollinearity. Finally, to explore the ideal number of factors retained in our Dutch sample we performed several factor analyses as suggested by Osborne & Costello (2005): (a) the four factor solution underlying the original PFS data was evaluated; (b) the factor solution suggested by the scree-test was evaluated; (c) if a and b resulted in different factor solutions, all factors above and below the suggested factor solutions of a and b were evaluated as well. After rotation we compared the different factor solutions to select the solution that fits best to the Dutch sample.


1. *Field A. Discovering Statistics Using SPSS. Third edit. London: SAGE Publications Ltd; 2000. 821 p.*

2. *Osborne JW, Costello AB. Best Practices in Exploratory Factor Analysis: Four Recommendations for Getting the Most From Your Analysis. Pract Assess Res Eval. 2005;10(7).*

3. *De Vet, Henrica C. W. Terwee CB, Knol DL, Mokkink LB. Measurement in Medicine. first. Cambridge: Cambridge University Press; 2011. 347 p.*
