# Supplementary material for: Translation and validation of the Dutch Pittsburgh Fatigability Scale for older adults
Source: BMC Geriatr. 2020 Jul 8;20:234. doi: 10.1186/s12877-020-01630-8 (PMC7346360; doi:10.1186/s12877-020-01630-8)
Supplement: Supplementary file 2 — Additional file 2. Supplementary figures and Tables. A flowchart of study participants and additional descriptive tables. [file 12877_2020_1630_MOESM2_ESM.pdf]

ADDITIONAL FILE 2: Supplementary figures and tables.

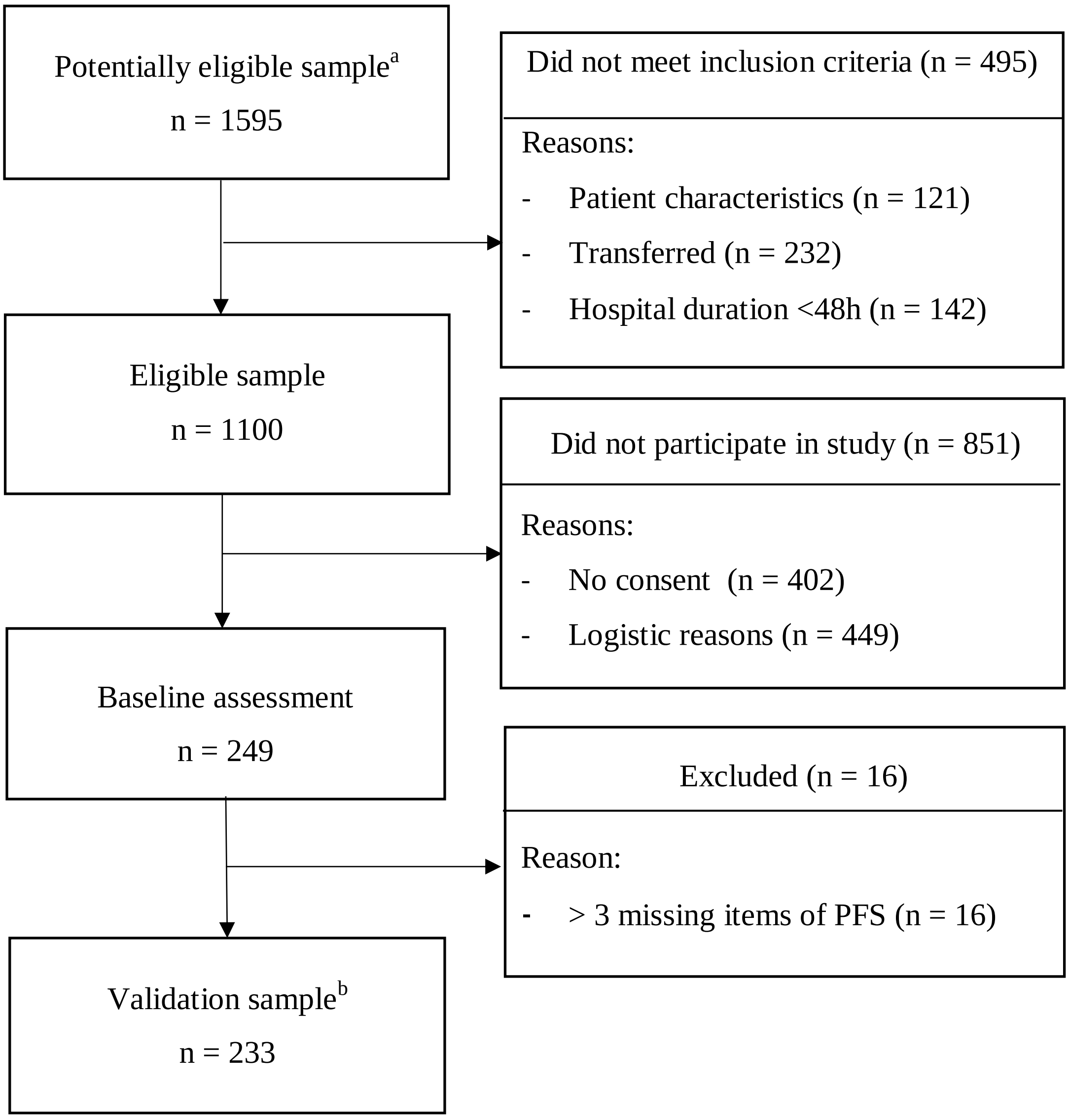

Figure S2 - 1: Flowchart of study participants

[a.] Hospitalized patients aged 70 years and older admitted to one of the participating wards
[b.] A random sample (n = 50) of the validation sample participated in the reliability sample

Table S2 -1a: Descriptive statistics for the items of the PFS physical subscale (n = 249)

**Original Subscales**		**Items**	**Mean (SD)**	**Median**	**Skewness (SE)**	**Kurtosis (SE)**	**Activity performed# (n (%))**	**Missing (n (%))**
moderate to high intensity activity	b.	Brisk or fast walk for 1 hour	3.73 (1.58)	4.00	-1.060 (0.158)	-0.044 (0.314)	70 (29)	11 (4)
	d.	Heavy gardening or outdoor work for 1 hour	3.39 (1.83)	4.00	-0.783 (0.158)	-0.844 (0.314)	102 (42)	11 (4)
	g.	Moderate- to high- intensity strength training for 30 min.	3.92 (1.42)	5.00	-1.357 (0.160)	0.969 (0.320)	52 (21)	19 (8)
	j.	High-intensity activity for 30 min.	3.91 (1.60)	5.00	-1.347 (0.160)	0.566 (0.319)	61 (26)	18 (7)
Social activity	h.	Participating in a social activity for 1 hour	1.26 (1.61)	0.00	1.002 (0.156)	-0.239 (0.311)	184 (76)	6 (2)
	i.	Hosting a social event for 1 hour	1.92 (1.85)	2.00	0.390 (0.156)	-1.307 (0.312)	133 (53)	7 (3)
Sedentary activity	e.	Watching TV for 2 hours	0.73 (1.25)	0.00	1.669 (0.157)	1.859 (0.312)	218 (90)	8 (3)
	f.	Sitting quietly for 1 hour	0.59 (1.15)	0.00	1.963 (0.156)	2.979 (0.310)	231 (96)	5 (2)
Lifestyle or light intensity activity	a.	Leisurely walk for 30 min.	2.59 (1.86)	3.00	-0.168 (0.156)	-1.339 (0.310)	141 (58)	5 (2)
	c.	Light household activity for 1 hour	1.90 (1.76)	2.00	0.394 (0.155)	-1.167 (0.309)	189 (77)	2 (1)

Number and valid percentage of patients who reported to perform the activity in the past month.

Abbreviations: SE, standard error; SD, standard deviation

Table S2- 1b: Descriptive statistics for the items of the PFS mental subscale

**Original Subscales**		**Items**	**Mean (SD)**	**Median**	**Skewness (SE)**	**Kurtosis (SE)**	**Missing (n (%))**
moderate to high intensity activity	b.	Brisk or fast walk for 1 hour	1.86 (1.97)	1.00	0.474 (0.158)	-1.365 (0.314)	11 (4)
	d.	Heavy gardening or outdoor work for 1 hour	1.78 (1.24)	1.00	0.566 (0.158)	-1.328 (0.316)	13 (5)
	g.	Moderate- to high- intensity strength training for 30 minutes	2.23 (2.03)	2.00	0.171 (0.160)	-1.602 (0.319)	18 (7)
	j.	High-intensity activity for 30 minutes	2.34 (2.08)	2.00	0.092 (0.161)	-1.649 (0.320)	20 (8)
Social activity	h.	Participating in a social activity for 1 hour	1.12 (1.54)	0.00	1.154 (0.156)	0.127 (0.310)	4 (2)
	i.	Hosting a social event for 1 hour	1.65 (1.79)	1.00	0.556 (0.156)	-1.161 (0.312)	7 (3)
Sedentary activity	e.	Watching TV for 2 hours	0.73 (1.24)	0.00	1.572 (0.157)	1.404 (0.312)	8 (3)
	f.	Sitting quietly for 1 hour	0.57 (1.14)	0.00	2.026 (0.156)	3.392 (0.310)	4 (2)
Lifestyle or light intensity activity	a.	Leisurely walk for 30 minutes	1.28 (1.71)	0.00	0.976 (0.156)	-0.507 (0.312)	7 (3)
	c.	Light household activity for 1 hour	1.15 (1.68)	0.00	1.191 (0.155)	-0.014 (0.309)	3 (1)

Abbreviations: SD, standard deviation; SE, standard error
