# Supplementary material for: Translation and validation of the Dutch Pittsburgh Fatigability Scale for older adults
Source: BMC Geriatr. 2020 Jul 8;20:234. doi: 10.1186/s12877-020-01630-8 (PMC7346360; doi:10.1186/s12877-020-01630-8)
Supplement: Supplementary file 3 — Additional file 3. Subgroup and sensitivity analyses. The results of the subgroup and sensitivity analyses. [file 12877_2020_1630_MOESM3_ESM.pdf]

ADDITIONAL FILE 3: Results subgroup and sensitivity analyses

Inhoud

Results subgroup analysis elective and acute patients..2

EFA PFS physical subscale 3

EFA PFS mental subscale...4

Results subgroup analysis surgical and non-surgical patients...7

EFA PFS physical subscale 8

EFA PFS mental subscale...9

Results sensitivity analysis using non-imputed data...12

Descriptive statistics and patient characteristics 12

Validity and EFA...13

Reliability...15

Results subgroup analysis elective and acute patients

Table S3 - 1: Baseline sociodemographic and patient characteristics of total group and subgroups.

Characteristic	Total (n=233)	Elective (n = 92)	Acute (n = 97)	Unknown (n = 44)
Age in years, median (IQR)	76 (73; 81)	76 (72; 81)	76 (73; 82)	77 (72; 83)
range	70 – 95	70 – 89	70 – 95	70 - 87
Sex, male	154 (66)	60 (65)	69 (71)	25 (57)
Educational level				
low (< high school)	61 (26)	24 (26)	21 (22)	16 (36)
moderate (high school)	84 (36)	33 (36)	35 (36)	16 (36)
high (college / university)	83 (36)	33 (36)	40 (41)	10 (23)
Medical specialism				
trauma and orthopedics	17 (7)	6 (7)	9 (9)	2 (4)
vascular and hepatobiliary	37 (16)	19 (21)	9 (9)	9 (21)
medical oncology	40 (17)	20 (22)	11 (11)	9 (21)
internal medicine	20 (9)	4 (4)	6 (6)	10 (23)
cardiology	114 (50)	43 (47)	62 (64)	9 (21)
Comorbidity[a], median (IQR)	2 (1; 3)	2 (1; 3)	2 (1; 3)	unknown
range	0 – 10	0 – 8	0 – 10	
LoS in days, median (IQR)	4 (3; 8)	4 (3; 6)	5 (3; 11)	unknown
range	1 – 37	1 – 30	1 – 37	
Fatigability score				
physical, mean (SD)	23.7 (11.5)	21.6 (11.2)	23.6 (11.2)	28.4 (11.4)
mental, mean (SD)	14.9 (13.5)	11.8 (12.1)	14.9 (13.2)	21.4 (14.7)

Notes: All numbers are presented in n (%), unless indicated otherwise.
Abbreviations: IQR, interquartile range; LoS, length of stay; SD, standard deviation.
a. Comorbidity score was assessed using the Charlson Comorbidity Index

***TableS3 - 2*:** Expected and observed correlations of the PFS subscales per subgroup with other constructs.

	Spearman's rank correlation			
**Instrument (scale range)**	PFS physical score (0-50)		PFS mental score (0-50)	
**Elective patients**	Expected	Observed	Expected	Observed
iADL (0-9)	0.3 – 0.5	0.3	0.3 – 0.5	0.2
Frailty Phenotype (0-5)	0.3 – 0.5	0.6	0.3 – 0.5	0.3
SBT (0-28)	≤ 0.3	0.04	≤ 0.3	0.04
**Acute patients**				
iADL (0-9)	0.3 – 0.5	0.5	0.3 – 0.5	0.3
Frailty Phenotype (0-5)	0.3 – 0.5	0.6	0.3 – 0.5	0.5
SBT (0-28)	≤ 0.3	0.1	≤ 0.3	0.1

Abbreviations: iADL, instrumental activities of daily living; PFS, Pittsburgh fatigability scale; SBT, short blessed test.

EFA PFS physical subscale

Kaiser-Meyer-Olkin (KMO) test and Bartlett's test of sphericity were performed for both subgroups, to check whether the data were suitable for EFA. KMO of the PFS physical subscale was 0.80 and 0.85 for elective and acute patients respectively, and Bartlett's test of sphericity was >0.001 for both subgroups, indicating that EFA could be applied.

Inspection of the scree-test and based on eigenvalues above 1 opt for a two-factor solution of both elective and acutely admitted patients with corresponding eigen values (explained variances): 4.55 (45%) plus 1.82 (18%), and 5.10 (51%) plus 1.72 (17%), respectively. Results of the two-factor solutions for both subgroups are presented in Table S3 - 3. Among elective patients the 2-factor solution resulted in a clean factor structure with all factor

loadings > 0.5. The first factor included items (A, B, C, D, G, and J) that required physical effort to perform. The second factor included the four items (E, F, H, and I) that require less physical activity, For acutely admitted patients one crossloading exists for household activities (item C). Cronbach's alphas indicated good internal consistency of the two factors for both subgroups.

EFA PFS mental subscale

KMO of the PFS mental subscale was 0.82 and 0.88 for elective and acute patients respectively, and Bartlett's test of sphericity was >0.001 for both subgroups, indicating that EFA could be applied.

The scree test and eigenvalues above 1 opt for a two-factor solution underlying the PFS mental subscale as well. Eigen values (% explained variance) were 5.55 (55%) plus 1.23 (12%), and 5.79 (58%) and 1.09 (11%) for elective and acutely admitted patients respectively. Results of the two-factor solution of the PFS mental subscale are presented in Table S3 - 4. Among elective patients, a crossloadings exists for item I (hosting a social event for one hour). Among acutely admitted patients, a crossloading existed for item C (light household activity for one hour). Cronbach's alphas indicated good internal consistency of the two factors for both subgroups.

Table S3 - 3: EFA results: Two-factor solutions for the Dutch version of the PFS physical subscale of elective and acutely admitted patients

		Elective		Acute	
		**Factor 1**	**Factor 2**	**Factor 1**	**Factor 2**
**Factor / item description**		α =0.89[a]	α =0.75[a]	α = 0.90[a]	α = 0.81[a]
**Moderate to high intensity activities**					
B	Brisk walking	**.947**	-	**.870**	-
D	Heavy gardening	**.792**	-	**.697**	-
G	Strength training	**.708**	-	**.820**	-
J	High intensity activity	**.709**	-	**.875**	-
**Lifestyle or light intensity activities**					
C	Household activity	**.517**	-	**.377**	**.532**
A	Leisurely walk	**.797**	-	**.681**	-
**Social activities**					
H	Participate in social activity	-	**.751**	-	**.767**
I	Hosting social event	-	**.655**	-	**.818**
**Sedentary activities**					
E	Watching TV	-	**.655**	-	**.655**
F	Sitting quietly	-	**.541**	-	**.585**

Only factor loadings >0.3 are presented.

a. Cronbach's alpha's were calculated for items A, B, C, D, G, J (Factor 1) and items E, F, H, I (Factor 2)

Table S3 - 4: EFA results: Two-factor solutions for the Dutch version of the PFS mental subscale of elective and acutely admitted patients.

		Elective		Acute	
		**Factor 1**	**Factor 2**	**Factor 1**	**Factor 2**
**Factor / item description**		α =0.91[a]	α =0.74[a]	α = 0.92[a]	α = 0.80[a]
**Moderate to high intensity activities**					
B	Brisk walking	**.952**	-	**.943**	-
D	Heavy gardening	**.807**	-	**.850**	-
G	Strength training	**.767**	-	**.642**	-
J	High intensity activity	**.649**	-	**.639**	-
**Lifestyle or light intensity activities**					
C	Household activity	**.578**	-	**.499**	**.336**
A	Leisurely walk	**.873**	-	**.927**	-
**Social activities**					
H	Participate in social activity	-	**.593**	-	**.754**
I	Hosting social event	**.512**	**.408**	-	**1.006**
**Sedentary activities**					
E	Watching TV	-	**.519**	-	**.489**
F	Sitting quietly	-	**.583**	-	**.460**

a. Cronbach's alpha's were calculated for items A, B, C, D, G, J (Factor 1) and items E, F, H, I (Factor 2)

Results subgroup analysis surgical and non-surgical patients

Table S3 - 5: Baseline sociodemographic and patient characteristics of total group and subgroups.

Characteristic	Total (n=233)	Surgical (n = 142)	Non-surgical (n = 60)	Unknown (n = 44)
Age in years, median (IQR)	76 (73; 81)	77 (73; 81)	76 (72; 82)	77 (72; 83)
range	70 – 95	70 – 93	70 – 95	70 - 87
Sex, male	154 (66)	92 (70)	37 (65)	25 (57)
Educational level				
low (< high school)	61 (26)	36 (27)	9 (16)	16 (36)
moderate (high school)	84 (36)	46 (35)	22 (39)	16 (36)
high (college / university)	83 (36)	47 (36)	26 (46)	10 (23)
Medical specialism				
trauma and orthopedics	17 (7)	8 (6)	7 (12)	2 (4)
vascular and hepatobiliary	37 (16)	20 (15)	8 (14)	9 (21)
medical oncology	40 (17)	24 (18)	7 (12)	9 (21)
internal medicine	20 (9)	1 (1)	9 (16)	10 (23)
cardiology	114 (50)	79 (60)	26 (46)	9 (21)
Comorbidity[a], median (IQR)	2 (1; 3)	2 (1; 3)	2 (1; 3)	unknown
range	0 – 10	0 – 9	0 – 10	
LoS in days, median (IQR)	4 (3; 8)	4 (3; 7)	4 (3; 9)	unknown
range	1 – 37	1 – 37	1 – 31	
Fatigability score				
physical, mean (SD)	23.7 (11.5)	21.8 (11.2)	24.6 (11.3)	28.4 (11.4)
mental, mean (SD)	14.9 (13.5)	12.9 (12.1)	14.5 (14.2)	21.4 (14.7)

Notes: All numbers are presented in n (%), unless indicated otherwise.
Abbreviations: IQR, interquartile range; LoS, length of stay; SD, standard deviation.
a. Comorbidity score was assessed using the Charlson Comorbidity Index

Table S3 - 6: Expected and observed correlations of the PFS subscales per subgroup with other constructs.

	**Spearman's rank correlation**			
**Instrument (scale range)**	PFS physical score (0-50)		PFS mental score (0-50)	
**Non surgical patients (n = 60)**	Expected	Observed	Expected	Observed
iADL (0-9)	0.3 – 0.5	0.4	0.3 – 0.5	0.1
Frailty Phenotype (0-5)	0.3 – 0.5	0.5	0.3 – 0.5	0.4
SBT (0-28)	≤ 0.3	0.1	≤ 0.3	0.04
**Surgical patients (n = 142)**				
iADL (0-9)	0.3 – 0.5	0.4	0.3 – 0.5	0.3
Frailty Phenotype (0-5)	0.3 – 0.5	0.6	0.3 – 0.5	0.4
SBT (0-28)	≤ 0.3	0.1	≤ 0.3	0.1

Abbreviations: iADL, instrumental activities of daily living; PFS, Pittsburgh fatigability scale; SBT, short blessed test.

EFA PFS physical subscale

Kaiser-Meyer-Olkin (KMO) test and Bartlett's test of sphericity were performed for both subgroups, to check whether the data were suitable for EFA. KMO of the PFS physical subscale was 0.81 and 0.83 for non-surgical and surgical patients respectively, and Bartlett's test of sphericity was >0.001 for both subgroups, indicating that EFA could be applied.

Inspection of the scree-test and based on eigenvalues above 1 opt for a two-factor solution of both non-surgical and surgical patients with corresponding eigen values (explained variances): 4.72 (47%) plus 2.03 (20%), and 4.86 (49%) plus 1.63 (16%), respectively. Results of the two-factor solutions for both subgroups are presented in Table S3 - 7. Among both non-surgical and surgical patients the 2-factor solution resulted in a clear factor structure

with all items loadings > 0.5 on one factor, except for the item household activities (item C), which show a crossloading. Theoretically we can distinguish the first factor that includes items that required physical effort to perform (A, B, C, D, G, and J). The second factor included items that require less physical activity (E, F, H, and I). An explanation why household activities load on both factors may be that depending on what type of household activity is thought of by answering; cooking can be considered as a predominantly mental activity, whereas vacuuming and cleaning windows requires physical effort to perform. Cronbach's alphas including household activity in the first factor indicated good internal consistency of the two factors for both subgroups.

EFA PFS mental subscale

KMO of the PFS mental subscale were 0.87 and 0.85 for non-surgical and surgical patients respectively, and Bartlett's test of sphericity was >0.001 for both subgroups, indicating that EFA could be applied.

The scree test and eigenvalues above 1 opt for a one factor solution underlying the PFS physical subscale data among non-surgical patients. Corresponding eigenvalue (6.31) explained 63% of the variance. Surgical patient data opt for a two-factor solution based on eigenvalues above 1 and scree test. Eigen values (% explained variance) were 5.31 (53%) plus 1.26 (13%) for surgical patients. Results of the factor solutions of the PFS mental subscale of non-surgical and surgical patients are presented in Table S3 - 8. All factor loadings of non-surgical patients were higher than 0.5 indicating a clean factor structure of the single factor solution. Exploring the two-factor solution for non-surgical patients resulted in an unexplainable cross-loading of item G (moderate to high intensity strength training). Among surgical patients, the two-factor solution resulted in a clean factor structure with six items (A, B, C, D, G, J) loading to the first factor and four items (E, F, H, I) loading to the second factor. All factor loadings were higher than 0.5 and no cross loadings indicating a clean factor

structure. Cronbach's alphas indicated good internal consistency of the single factor solution for non-surgical patients and the two factor solution for surgical patients.

Table S3 - 7: EFA results: Two-factor solutions for the Dutch version of the PFS physical subscale of non-surgical and surgical patients (pattern matrix).

		Non-surgical		Surgical	
		**Factor 1**	**Factor 2**	**Factor 1**	**Factor 2**
**Factor / item description**		α =0.88[a]	α =0.83[a]	α = 0.90[a]	α = 0.75[a]
**Moderate to high intensity activities**					
B	Brisk walking	**.810**	-	**.953**	-
D	Heavy gardening	**.707**	-	**.749**	-
G	Strength training	**.781**	-	**.755**	-
J	High intensity activity	**.869**	-	**.757**	-
**Lifestyle or light intensity activities**					
C	Household activity	**.443**	**.460**	**.449**	**.370**
A	Leisurely walk	**.684**	-	**.784**	-
**Social activities**					
H	Participate in social activity	-	**.824**	-	**.751**
I	Hosting social event	-	**.784**	-	**.727**
**Sedentary activities**					
E	Watching TV	-	**.649**	-	**.652**
F	Sitting quietly	-	**.660**	-	**.508**

a. Cronbach's alpha's were calculated for items A, B, C, D, G, J (Factor 1) and items E, F, H, I (Factor 2)

Table S3 - 8: EFA results: Two-factor solutions for the Dutch version of the PFS mental subscale of non-surgical and surgical patients (pattern matrix).

		Non-surgical	Surgical	
		**Factor 1**	**Factor 1**	**Factor 2**
**Factor / item description**		α =0.94[a]	α = 0.91[b]	α = 0.74[b]
**Moderate to high intensity activities**				
B	Brisk walking	**.852**	**.965**	-
D	Heavy gardening	**.848**	**.705**	-
G	Strength training	**.819**	**.653**	-
J	High intensity activity	**.803**	**.594**	-
**Lifestyle or light intensity activities**				
C	Household activity	**.766**	**.574**	-
A	Leisurely walk	**.832**	**.903**	-
**Social activities**				
H	Participate in social activity	**.863**	-	**.744**
I	Hosting social event	**.828**	-	**.757**
**Sedentary activities**				
E	Watching TV	**.592**	-	**.457**
F	Sitting quietly	**.559**	-	**.486**

a. Cronbach's alpha was calculated for all ten items

b. Cronbach's alpha's were calculated for items A, B, C, D, G, J (Factor 1) and items E, F, H, I (Factor 2)

Results sensitivity analysis using non-imputed data

Descriptive statistics and patient characteristics

Table S3 - 9: Baseline characteristics of total group and subgroups.

Characteristic	Total (n=233)	Complete PFS data (n = 201)
Age in years, median (IQR)	76 (73; 81)	76 (73; 82)
range	70 – 95	70 – 95
Sex, male	154 (66)	137 (68)
Educational level		
low (< high school)	61 (26)	51 (25)
moderate (high school)	84 (36)	73 (36)
high (college / university)	83 (36)	72 (36)
Medical specialism		
trauma and orthopedics	17 (7)	15 (8)
vascular and hepatobiliary	37 (16)	31 (15)
medical oncology	40 (17)	29 (14)
internal medicine	20 (9)	19 (10)
cardiology	114 (50)	103 (51)
Comorbidity[a], median (IQR)	2 (1; 3)	2 (1; 3)
range	0 – 10	0 – 10
LoS in days, median (IQR)	4 (3; 8)	4 (3; 8)
range	1 – 37	1 – 37
Fatigability score		
physical, mean (SD)	23.7 (11.5)	24.0 (11.5)
mental, mean (SD)	14.9 (13.5)	15.4 (13.6)

Notes: All numbers are presented in n (%), unless indicated otherwise.
Abbreviations: IQR, interquartile range; LoS, length of stay; SD, standard deviation.
a. Comorbidity score was assessed using the Charlson Comorbidity Index

Validity and EFA

***Table S3 - 10*: **Expected and observed correlations of the PFS subscales of patients with complete PFS data (n = 201).

**Instrument (scale range)**	**Spearman's rank correlation**			
	PFS physical score (0-50)		PFS mental score (0-50)	
	Expected	Observed	Expected	Observed
iADL (0-9)	0.3 – 0.5	0.4	0.3 – 0.5	0.3
Frailty Phenotype (0-5)	0.3 – 0.5	0.5	0.3 – 0.5	0.4
SBT (0-28)	≤ 0.3	0.05	≤ 0.3	0.05

Abbreviations: iADL, instrumental activities of daily living; PFS, Pittsburgh fatigability scale; SBT, short blessed test.

Table S3 - 11: EFA results: Pattern matrix of the 2 factor solutions of the PFS physical and mental subscale of the patients with complete PFS data (sensitivity analysis) (n=201)

		Physical subscale		Mental subscale	
**Factor / Item description**		**Factor 1**	**Factor 2**	**Factor 1**	**Factor 2**
		**(α: 0.89)[a]**	**(α: 0.81)[a]**	**(α: 0.93)[a]**	**(α: 0.81)[a]**
**Moderate to high intensity activities**					
B	Brisk walking	**.923**	-	**.985**	-
D	Heavy gardening	**.740**	-	**.801**	-
G	Strength training	**.824**	-	**.629**	-
J	High intensity activity	**.794**	-	**.591**	-
**Lifestyle or light intensity activities**					
C	Household activity	**.368**	**.515**	**.520**	**.337**
A	Leisurely walk	**.640**	-	**.900**	-
**Social activities**					
H	Participate in social activity	-	**.799**	-	**.790**
I	Hosting social event	-	**.730**	-	**.744**
**Sedentary activities**					
E	Watching TV	-	**.727**	-	**.640**
F	Sitting quietly	-	**.601**	-	**.594**

Factors loaded >0.3 are presented

Principal-Axis Factor analysis was used with obligue oblimin rotation

a. Cronbach's Alpha of the PFS physical subscale was calculated using items A, B, C, D, G, J and items E, F, H, I for factors 1 and 2, respectively.

Reliability

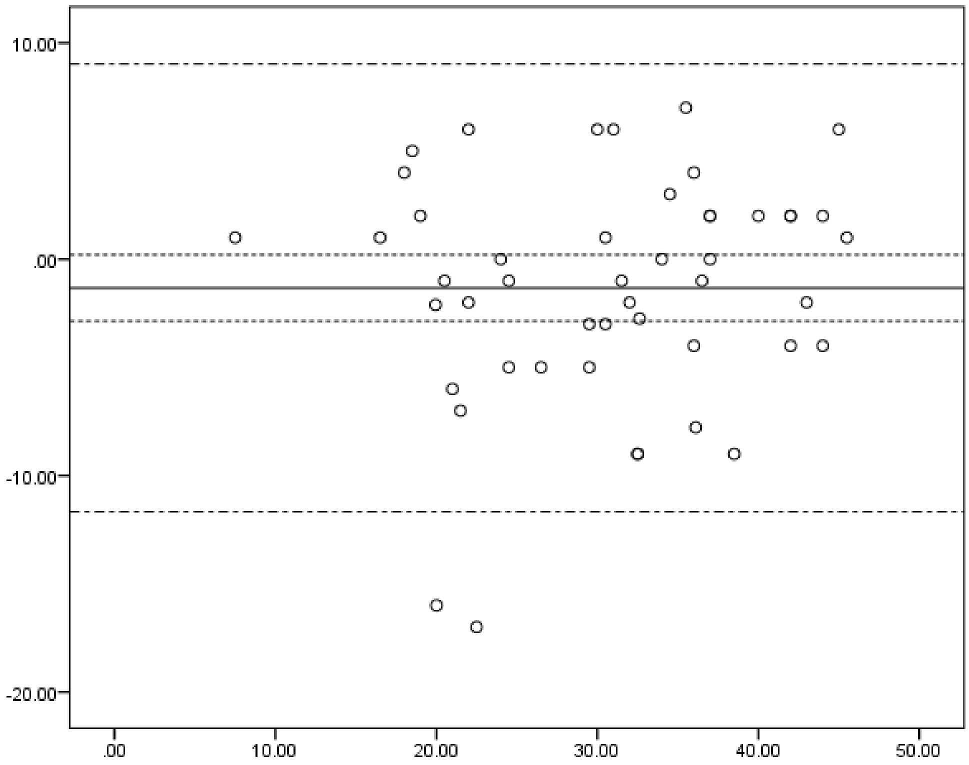

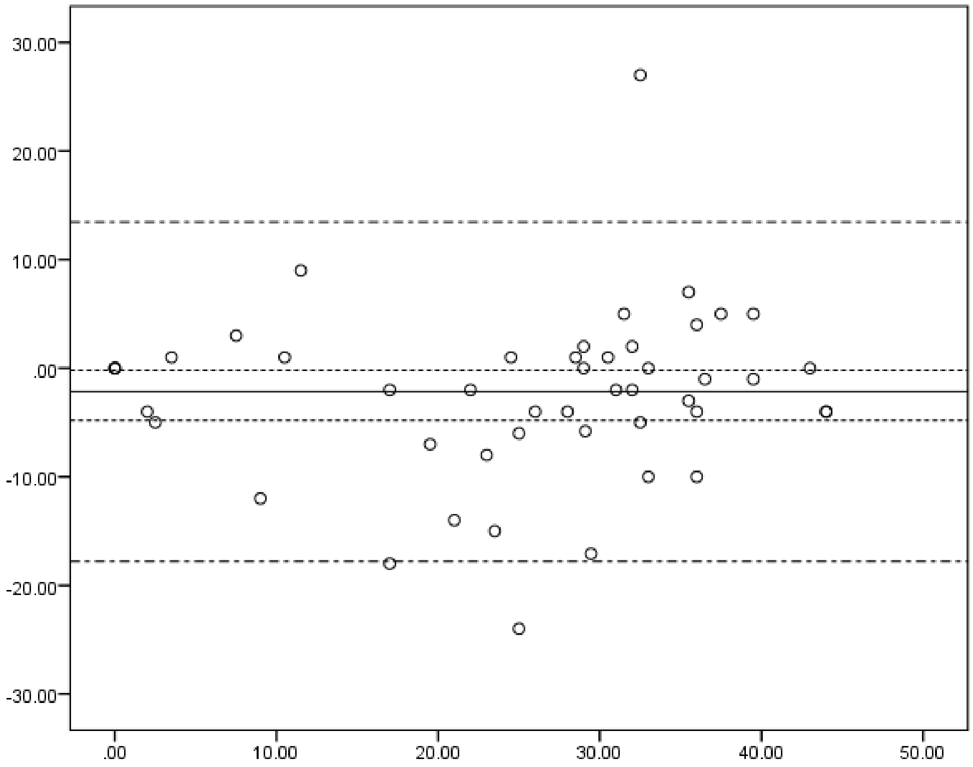

Figure S3 -1: Bland Altman plots of the PFS physical (a) and mental (b) subscales (sensitivity analysis). X-axis represent mean fatigability scores; Y-axis represents difference of fatigability scores (baseline – retest assessments).

Bland Altman plots showed good agreement for the PFS physical subscale (mean difference -1.33, 95% CI: -2.85; 0.21). The PFS mental subscale had a small mean systematic difference between baseline and re-assessment (mean difference: -2.50, 95% CI: -4.81; -0.18). Reliability outcomes are presented in table 4. ICC of the PFS physical and mental subscales were 0.84 (95% CI: 0.73; 0.91) and 0.81 (95% CI: 0.67; 0.89), respectively, indicating good reliability.

***Table S3 - 12*:** Mean scores and standard deviations of baseline and retest assessments (n =

**PFS**	**Baseline mean (SD)**	**T1 mean (SD)**	**ICC (95% CI)**	**SEM**	**SEM (%)[a]**	**SDC**	**SDC (%)[a]**
Physical subscale	30 (10)	31 (9)	0.84 (0.73; 0.91)	4	8%	11	22%
Mental subscale	24 (13)	26 (13)	0.81 (0.67; 0.89)	6	12%	17	33%

Abbreviations: PFS, Pittsburgh Fatigability Scale; ICC, intraclass correlation coefficient for agreement using a 2 way mixed effect model; SEM, standard error of measurement; SDC, smallest detectable change; MIC, minimal important change.

a. SEM (%) and SDC (%) are expressed in percentages of the scale range (0-50); percentages are rounded off.
